# Antioxidative Diet Supplementation Reverses High-Fat Diet-Induced Increases of Cardiovascular Risk Factors in Mice

**DOI:** 10.1155/2015/467471

**Published:** 2015-04-01

**Authors:** Hilda Vargas-Robles, Amelia Rios, Monica Arellano-Mendoza, Bruno A. Escalante, Michael Schnoor

**Affiliations:** ^1^Department for Molecular Biomedicine, Center of Research and Advanced Studies (CINVESTAV) of the National Polytechnic Institute (IPN), Zacatenco, 07360 Mexico City, DF, Mexico; ^2^CINVESTAV-IPN Monterrey, 66600 Apodaca, NL, Mexico; ^3^Superior Medical School, IPN, Postgraduate and Research Section, 11340 Mexico City, DF, Mexico

## Abstract

Obesity is a worldwide epidemic that is characterized not only by excessive fat deposition but also by systemic microinflammation, high oxidative stress, and increased cardiovascular risk factors. While diets enriched in natural antioxidants showed beneficial effects on oxidative stress, blood pressure, and serum lipid composition, diet supplementation with synthetic antioxidants showed contradictive results. Thus, we tested in C57Bl/6 mice whether a daily dosage of an antioxidative mixture consisting of vitamin C, vitamin E, L-arginine, eicosapentaenoic acid, and docosahexaenoic acid (corabion) would affect cardiovascular risk factors associated with obesity. Obese mice showed increased serum triglyceride and glucose levels and hypertension after eight weeks of being fed a high-fat diet (HFD). Importantly, corabion ameliorated all of these symptoms significantly. Oxidative stress and early signs of systemic microinflammation already developed after two weeks of high-fat diet and were significantly reduced by daily doses of corabion. Of note, the beneficial effects of corabion could not be observed when applying its single antioxidative components suggesting that a combination of various nutrients is required to counteract HFD-induced cardiovascular risk factors. Thus, daily consumption of corabion may be beneficial for the management of obesity-related cardiovascular complications.

## 1. Introduction

Obesity and obesity-related secondary pathologic conditions such as metabolic syndrome, cardiovascular diseases (CVD), diabetes, and cancer are serious health conditions and constitute a huge burden for the public health systems worldwide [1]. Metabolic syndrome (MetS) is a serious consequence of obesity characterized by increased cardiovascular risk factors such as hypertension, dyslipidemia, and glucose intolerance. The main causes of obesity and MetS are high-energy diet and lack of physical activity [2]. However, although consumption of low-fat products is generally recommended, consumption of high-fat dairy products, for example, does not seem to be related to a higher risk of obesity and CVD [3]. Increasing evidence is now being published that oxidative stress is also an important feature of high-fat diet (HFD)-induced MetS responsible for vascular damage and increased risk of CVD [4]. For example, early phases of obesity are characterized by increased production of reactive oxygen species (ROS), decreased NO bioavailability, and consequently endothelial dysfunction [5]. Thus, diets enriched in components reducing oxidative stress are under investigation. Consumption of foods rich in antioxidants such as certain fruits, legumes, nuts, and vegetables has been shown to counteract obesity and MetS [6–8]. However, even though a well-balanced diet seems to be the key factor for preventing obesity and its risks, it is hard, if not impossible, for most people to stick to such diets. Thus, antioxidative supplements have garnered some attention with respect to the management of obesity and MetS [9]. So far, contradictive results have been published from human and murine studies testing various antioxidants. Two of the most important antioxidative micronutrients are vitamin C and vitamin E [4]. In particular, vitamin E supplementation has been found to lower plasma biomarkers of oxidative stress [10, 11].* In vitro*, vitamin E also showed atheroprotective properties by, for example, preventing oxidation of LDL; however this has not yet been observed in clinical trials (reviewed in [12, 13]). Vitamin C supplementation also reduced oxidative stress but not to the same extent as spinach, strawberries, or red wine suggesting that a combination of different antioxidants has additive beneficial effects on oxidative stress [14]. Indeed, using corabion (a combination of the antioxidative vitamins C and E, L-arginine (NO source), eicosapentaenoic acid (EPA), and docosahexaenoic acid (DHA)), we previously reported beneficial effects on NO bioavailability and kidney damage parameters during induced kidney failure in mice [15, 16]. Thus, the use of this combination as dietary supplement may constitute a simple, cheap, and healthy way to also counteract obesity-induced increases in CVD risk factors.

## 2. Methods

### 2.1. Mice, Induction of Obesity, and Antioxidative Treatment

All animal experiments were approved by the Institutional Animal Care and Use Committee of CINVESTAV, Mexico City. Male C57Bl/6 mice from the Animal Facility at CINVESTAV were used at an age of five weeks. Mice were housed under conditions of constant temperature, humidity, and standard dark-light cycles of 12 h with* ad libitum* access to food and water. Mice were randomly separated into the following groups: the control group (control) was fed a reference diet for 8 weeks (5058, Purina, St. Louis, MO, energy content: 21 kcal% fat, 24 kcal% protein, and 55 kcal% carbohydrates), the high-fat diet (HFD) group was fed a HFD for 8 weeks (D12331, Jackson Laboratory, Sacramento, CA, energy content: 58 kcal% fat, 16 kcal% protein, and 26 kcal% carbohydrates), and the antioxidant group was fed a HFD plus a mixture of 200 mg/kg L-arginine, 83 mg/kg vitamin C, 46.6 mg/kg vitamin E, 77 mg/kg eicosapentaenoic acid, and 115 mg/kg docosahexaenoic acid (corabion, kindly provided by Merck, Naucalpan, Mexico) dissolved in a 1 : 1 mixture of water and safflower oil given once daily by oral gavage (50 *μ*L). The data in Table 1 were generated by additionally using the following groups: HFD plus 200 mg/kg L-arginine (HFD + L-arginine), HFD plus 46.6 mg/kg vitamin E (HFD + vitamin E), and HFD plus 83 mg/kg vitamin C (HFD + vitamin C). Mice that did not receive corabion were sham gavage-fed daily with 50 *μ*L of a 1 : 1 water : safflower oil suspension (vehicle) to exclude effects of the feeding procedure. The single components L-arginine and vitamin E and C were also gavage-fed to ensure consumption of the proper dosage.

### 2.2. Measurement of Metabolic Parameters

Blood samples were taken from the tail after overnight starvation and triglycerides and glucose levels were measured using a glucometer (One Touch BASIC Plus, Johnson & Johnson, Milpitas, CA) and an Accu-Chek sensor (Roche Diagnostics, Mexico City, Mexico), respectively, according to the manufacturer's instructions.

### 2.3. Measurement of Biopterins

Blood was collected by cardiac puncture immediately after sacrificing the animals at the indicated time points. Reduced (tetrahydrobiopterin, BH4) and oxidized (dihydrobiopterin, BH2) forms of biopterins in mouse plasma were determined by capillary zone electrophoresis as described [15]. Before measurement using a P/ACETM MDQ electrophoresis system (Beckman Coulter, Mexico City, Mexico), plasma was deproteinized and filtered. Concentrations were calculated on the basis of a standard curve of biopterin diluted in 6 mM phosphate buffer (pH 7.4).

### 2.4. Fluorescence Microscopy of Oxidative Stress

Production of superoxides in the kidneys was measured by means of the fluorescence dye dihydroethidium (DHE, Life Technologies, Grand Island, NY) as described previously [17]. Briefly, 10 *μ*m thick tissue cross sections were collected on glass slides and 5 *μ*M DHE in water was added to each tissue section and incubated in a dark humidified chamber at 37°C for 30 minutes. DHE is oxidized to ethidium by superoxide anions that translocates to the nucleus where it intercalates with genomic DNA. Fluorescence of ethidium as evidence of ROS production was recorded using a laser scanning confocal imaging system (FV-300, Olympus, Miami, FL).

### 2.5. Blood Pressure Measurement

After body weight measurement, mice were anesthetized by intraperitoneal injection of 60 mg/kg pentobarbital and blood pressure was measured by cannulating the carotid artery with a polyethylene catheter (inner diameter 0.011 inches and outer diameter 0.24 inches, Clay Adams, Nutley, NJ, USA) connected to a solid state pressure transducer (DUO.18 WPI, Aston, UK) at the indicated time points (2, 4, and 8 weeks after the respective diets) before sacrificing the animals. Blood pressure was measured for 15 min.

### 2.6. RT-PCR

TRIzol (Invitrogen, Carlsbad, CA) was used according to the manufacturer's instructions to extract total RNA from whole kidney lysates. cDNA was produced by reverse transcription using oligo(dT_12-18_) primers and Superscript II (Invitrogen). PCRs were performed with Taq-DNA polymerase (Roche, Indianapolis, IN) on a Veriti 96-well thermal cycler (Applied Biosystems, Mexico City, Mexico). Primers were designed so that forward and reverse primers are located in different exons to prevent amplification of genomic DNA and obtained from Uniparts (Mexico City, Mexico). Mouse-specific primer sequences were TNF-a-FW: ACGGCATGGATCTCAAAGAC and TNF-a-RE: AGATAGCAAATCGGCTGACG; IL6-FW: CCTTCCTACCCCAATTTCCAA and IL6-RE: AGATGAATTGGATGGTCTTGGTC; *β*-actin-FW: TATCCACCTTCCAGCAGATGT and *β*-actin-RE: AGCTCAGTAACAGTCCGCCTA; GAPDH-FW: TGTCATACTTGGCAGGTTTCT and GAPDH-RE: CGTGTTCCTACCCCCAATGT. PCR conditions were 95°C for 5 min followed by 30 cycles of 95°C for 15 s, 60°C for 30 s, and 72°C for 45 s followed by a final extension at 72°C for 10 min. Amplicons were analyzed by 2% agarose gel electrophoresis.

### 2.7. Measurement of C-Reactive Protein (CRP)

Levels of CRP in mouse serum were determined using the CRP latex agglutination kit (Spinreact, Girona, Spain) according to the manufacturer's instructions.

### 2.8. Statistics

All data are expressed as means ± standard deviation of the means. Significance was evaluated using ANOVA followed by Tukey's test. *P* values less than 0.05 were considered statistically significant.

## 3. Results

### 3.1. HFD-Induced Obesity Increases CVD Risk Factors

**Figure 1:**
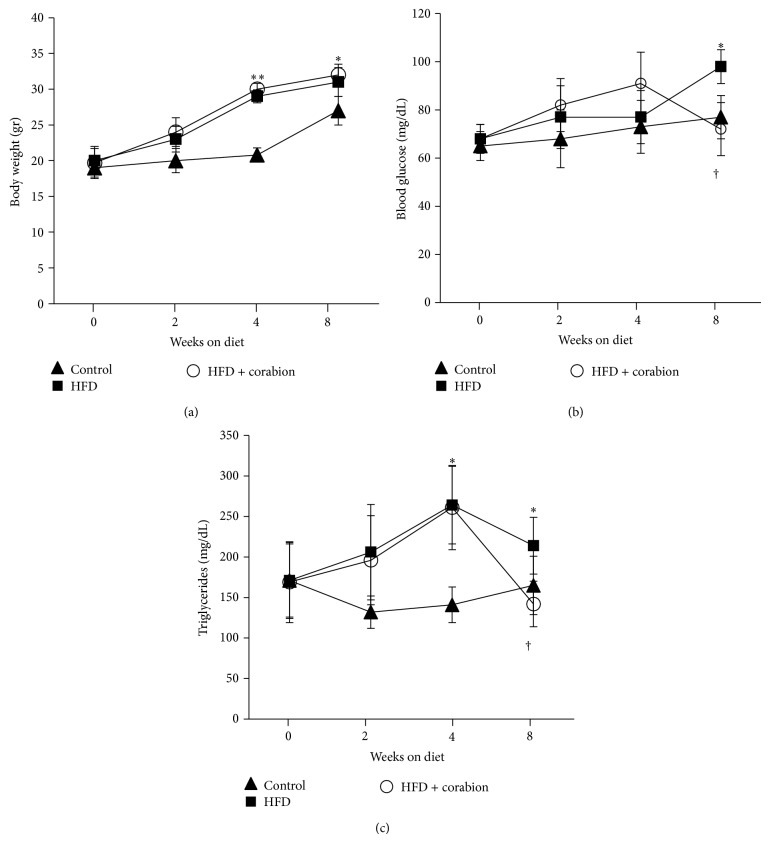
Antioxidative treatment reverses increases in HFD-induced blood glucose and triglycerides levels without affecting body weight. Mice were fed either a normal diet, a high-fat diet (HFD), or a HFD supplemented with corabion for 8 weeks starting at an age of 5 weeks. CVD risk factors were recorded at the indicated times: (a) body weight, (b) fasting blood glucose, and (c) plasma triglycerides. The *x*-axes indicate weeks after start of the respective diets. *n* = 10 for the control and HFD + corabion groups; *n* = 20 for the HFD group; ^∗^ *P* < 0.05; ^∗∗^ *P* < 0.01 (significance control versus HFD); ^†^ *P* < 0.05 (significance HFD versus HFD + corabion).

To investigate whether a HFD induces MetS in our experimental setting, we measured the metabolic parameters body weight, fasting blood glucose, and triglycerides over a period of eight weeks starting at an age of 5 weeks (Figure 1). In a previous study, we investigated the effects of early obesity on kidney damage and observed that a two-week period of HFD was not sufficient to induce MetS since at this time point triglyceride and glucose plasma levels and blood pressure were not yet altered significantly [16]. Our current time course confirmed these previous findings since, two weeks after starting the respective diets, we did not observe statistically significant differences for body weight (Figure 1(a)), blood glucose (Figure 1(b)), and triglycerides (Figure 1(c)). By contrast, four weeks of a HFD induced significant increases of body weight and triglycerides but not blood glucose. After eight weeks of HFD, all parameters were significantly increased (Figure 1) but the increase in triglycerides was less pronounced than after four weeks (Figure 1(c)). To exclude any effect of the different diets on the survival of these mice, we followed the development of extra groups for a period of one year and all animals survived this experimental period. Additionally, we used metabolic cages to monitor if food consumption changed due to the different diets. However, we did not observe significant differences in overall food consumption between the different diet groups (data not shown).

### 3.2. Antioxidative Diet Supplement Reduces HFD-Induced CVD Risk Factors

Given the fact that the antioxidative vitamins C and E as well as the NO source L-arginine have been shown to ameliorate obesity-induced kidney damage and CVD risk factors [4, 16, 18], we wanted to know whether a combination of these micronutrients in the formula corabion given by daily gavage during the whole experimental procedure could reverse obesity-induced CVD risk factors in mice. As expected, antioxidative treatment did not affect the gain in body weight over the experimental period of 8 weeks (Figure 1(a)). After two and four weeks of treatment, corabion did not significantly affect the levels of blood glucose and triglycerides. However, after eight weeks of HFD with daily antioxidative treatment, we observed significant lower levels of blood glucose (Figure 1(b)) and triglycerides (Figure 1(c)) compared to HFD alone. Interestingly, only the entire formula corabion, but not its single components, showed these beneficial effects after 8 weeks of treatment (Table 1), suggesting that a combination of various antioxidants is required to show beneficial effects on HFD-induced CVD risk factors.

### 3.3. HFD-Induced Obesity Increases Oxidative Stress

**Figure 2:**
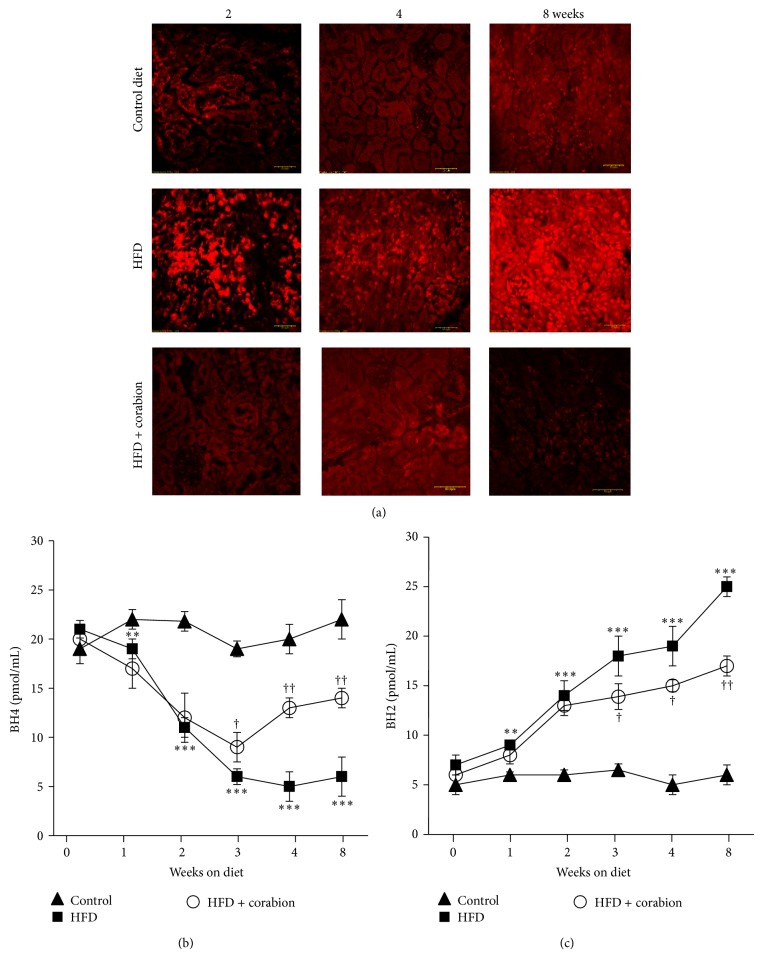
Antioxidative treatment completely reverses HFD-induced ROS production in kidneys and partially reverses oxidative stress in the circulation. Mice were fed either a normal diet, a high-fat diet (HFD), or a HFD supplemented with corabion for eight weeks starting at an age of five weeks. (a) Production of ROS as measure of oxidative stress was determined in 10 *μ*m kidney cryosections by DHE assays at the indicated times. Red fluorescence of ethidium in the nuclei is depicted. Bar = 50 *μ*m. (b) Levels of tetrahydrobiopterin (BH4) (c) and dihydrobiopterin (BH2) in plasma samples as measure of vascular oxidative stress were determined by capillary electrophoresis at the indicated times. The *x*-axes indicate weeks after start of the respective diets. *n* = 5; ^∗∗^ *P* < 0.01; ^∗∗∗^ *P* < 0.001 (significance control versus HFD); ^†^ *P* < 0.05; ^††^ *P* < 0.01 (significance HFD versus HFD + corabion).

Since oxidative stress is now considered an important feature of MetS, we sacrificed animals after two, four, and eight weeks of the respective diets and determined production of ROS in kidney cross sections. As can be seen in Figure 2(a), oxidative stress is strongly increased already after two weeks of HFD even though the other described parameters were still normal (cf. Figure 1). These data suggest that oxidative stress is among the first risk factors to change during development of obesity and MetS. The increase was less pronounced after four weeks but peaked after eight weeks. This result prompted us to investigate the ratio of BH4 to BH2, another marker of oxidative stress in the circulation, in the respective mice. To confirm the relevance of oxidative stress as one of the first risk factors to increase on a HFD, we sacrificed mice every week over the 8-week experimental period and measured BH4 and BH2 plasma levels by capillary electrophoresis. While the levels of BH4 (reduced form) were already significantly lower in HFD animals as early as one week after starting the diet (Figure 2(b)), the differences were even more pronounced after two and three weeks and then remained constant over the entire experimental period. Concomitantly, the levels of BH2 (oxidized form) rose constantly in HFD animals over the entire experimental period (Figure 2(c)). These data emphasize the importance of oxidative stress as a first marker of obesity-induced systemic changes.

### 3.4. Antioxidative Diet Supplement Reduces HFD-Induced Oxidative Stress

Examining oxidative stress in kidney cross sections, we saw a complete inhibition of ROS production in the HFD group supplemented with corabion when compared to the HFD group (Figure 2(a)). In fact, oxidative stress appeared to be even lower than the basal level of the control group, especially after 8 weeks of treatment. This result was confirmed by measuring the levels of BH4 and BH2 in animals fed a HFD with corabion. Obese animals showed low amounts of BH4 (Figure 2(b)) but high amounts of oxidized BH2 (Figure 2(c)). These changes induced by a HFD were significantly ameliorated as early as 3 weeks of corabion supplementation and this amelioration got more pronounced over the 8-week treatment period although the HFD-induced changes were not completely reversed (Figures 2(b) and 2(c)).

### 3.5. Antioxidative Diet Supplement Reduces HFD-Induced Increases in Blood Pressure

**Figure 3:**
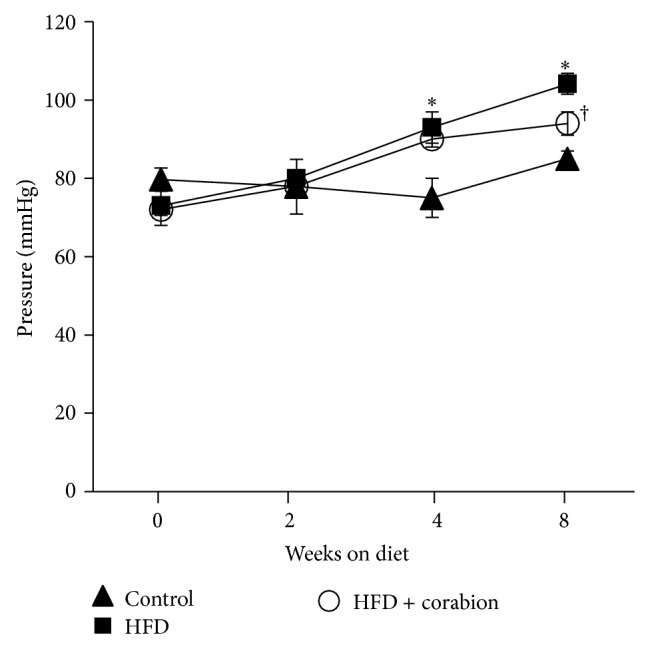
Antioxidative treatment counteracts HFD-induced blood pressure increase after 8 weeks. Mice were fed either a normal diet, a high-fat diet (HFD), or a HFD supplemented with corabion for eight weeks starting at an age of five weeks. Blood pressure was measured for 15 min on anaesthetized mice at the indicated times. The *x*-axes indicate weeks after start of the respective diets. *n* = 10 for the control and HFD + corabion groups; *n* = 20 for the HFD group; ^∗^ *P* < 0.05 (significance control versus HFD); ^†^ *P* < 0.05 (significance HFD versus HFD + corabion).

Increased blood pressure is another important CVD risk factor that is often increased during obesity. Thus, we wanted to know whether a HFD would increase blood pressure and whether corabion would be able to counteract these changes. Indeed, blood pressure was significantly increased after 4 and 8 weeks of a HFD (Figure 3). Corabion did not have an effect after 4 weeks but significantly counteracted the HFD-induced increase in blood pressure after 8 weeks.

### 3.6. HFD-Induced Systemic Microinflammation Is Ameliorated by the Antioxidative Diet Supplement

**Figure 4:**
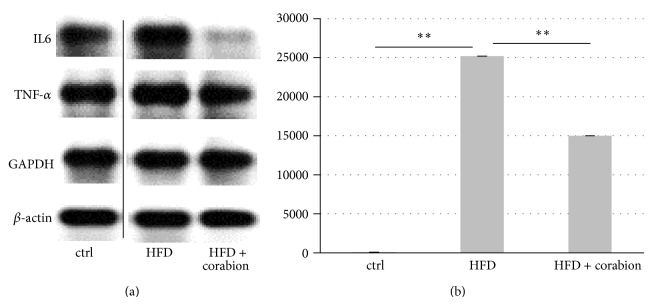
Antioxidative treatment partially reverses early signs of microinflammation after two weeks. Mice were fed either a control diet (ctrl), a high-fat diet (HFD), or a HFD with corabion for two weeks starting at an age of five weeks. (a) Production of IL6 and TNF mRNAs as measure of an early inflammatory response was determined by semiquantitative RT-PCR of cDNA derived from kidney of the respective mice.   *β-actin *and GAPDH were used as housekeeping gene. Representative images of triplicates from two animals per group are shown. (b) Levels of C-reactive protein (CRP) in blood serum samples, as independent inflammatory marker in the circulation, were determined by agglutination. Depicted are changes in percent compared to mice on control diet (ctrl set to 100%). *n* = 2 measured in the triplicates; ^∗∗^ *P* < 0.01.

Microinflammation, as manifested by increased production and secretion of proinflammatory cytokines, is a hallmark of obesity. Thus, we performed semiquantitative RT-PCR for interleukin-6 (IL6) and tumor necrosis factor-*α* (TNF-*α*) of cDNA derived from kidney and agglutination assays for C-reactive protein in mouse serum to determine if early systemic microinflammation occurs after 2 weeks of HFD. Interestingly, neither a HFD alone nor HFD with corabion led to significant changes of TNF-*α* mRNA in kidneys after two weeks of the respective diets (Figure 4(a)). By contrast, HFD led to a strong increase in IL6 mRNA synthesis in the kidney after 2 weeks, and corabion significantly counteracted this HFD-induced increase in IL6 mRNA production (Figure 4(a)). Since IL6 is known to induce CRP production in the liver and secretion into the blood, we screened for CRP levels in sera of mice on the respective diets (Figure 4(b)). While CRP was barely detectable in mice on a control diet (0.005 mg/dL), we detected a strong increase in CRP levels after 2 weeks of HFD (1.22 ± 0.056 mg/dL) corresponding to levels of early inflammation. Of note, corabion reduced the HFD-induced increase in serum CRP by more than 40% (0.71 ± 0.035 mg/dL) indicating that corabion alleviates not only HFD-induced oxidative stress but also early systemic microinflammatory responses.

## 4. Discussion

Here, we demonstrate that oxidative stress in the vasculature, as indicated by a shifted BH4-BH2 ratio, increases already after one week of a HFD and can thus be considered one of the first markers of beginning obesity and MetS. Moreover, we show that corabion is a beneficial diet supplement for the management of obesity-related cardiovascular risk factors. Interestingly, only this combination of nutrients but not treatment with single antioxidative corabion components showed beneficial effects on HFD-induced CVD risk factors (Table 1).

Oxidative stress is characterized by excessive formation or defective removal of reactive oxygen species (ROS), occurs during both obesity and MetS, and is responsible for, for example, endothelial dysfunction [9, 19]. CVD risk factors such as hyperglycemia have been shown to cause activation of NADPH oxidase and an increase in the production of ROS such as the superoxide anion (O_2_^−^) [20]. Concomitantly, reduced levels of nitric oxide (NO) have been observed to further deteriorate endothelial functionality and cardiovascular sanity during MetS [21]. Interestingly, oxidative stress in individuals suffering from hypertension, another important CVD risk factor, was not further affected by other risk factors such as high triglyceride and fasting glucose levels [22]. This would imply that hypertension is a risk factor for developing oxidative stress. On the other hand, our data clearly show that, under a HFD, oxidative stress develops before the onset of hypertension suggesting that oxidative stress is rather a risk factor for the development of hypertension. However, these observations may very well be species-specific differences and further research is needed to clarify this point.

Natural protection against oxidative stress is provided by enzymes that degrade ROS such as superoxide dismutase or catalase and by nonenzymatic compounds such as vitamins E and C. ROS overproduction causes a disbalance between the mentioned oxidative and antioxidative factors leading to oxidative stress. Indeed, MetS is characterized not only by oxidative stress but also by reduced vitamin E and C levels suggesting that diet supplementation with these vitamins could reverse oxidative stress and related risk factors [23]. The impact of vitamin supplementation on oxidative stress and CVD risk factors in MetS has been the focus of several studies with contradicting results. In particular, vitamin E supplementation reduced cholesterol levels and oxidative stress in Chinese women [24] and could prevent onset of type 2 diabetes [25]. Vitamin C improved the antioxidative capacity of serum in elderly women [14] and could also restore defective hyperglycemia-dependent vasodilation [26]. By contrast, another study failed to show beneficial effects of a daily vitamin E dosage on cardiovascular complications in humans [27]. Moreover, a combination of vitamins C and E did not affect CVD risk factors such as body weight, low density lipoprotein, or triglyceride concentrations in patients with established MetS [28]. In patients with coronary artery disease or type 2 diabetes, vitamin E also did not improve markers of oxidative stress [10, 11]. Furthermore, vitamin E failed to protect against atherosclerosis in Ldlr-deficient mice when fed a western-style HFD [29] and also did not show atheroprotective effects in clinical trials [12, 13]. These data imply that vitamin supplementation alone is not sufficient to improve established complications of obesity or MetS, whereas a combination of various antioxidative compounds, as in corabion, does reduce HFD-induced CVD risk factors. Our data may also help to explain why studies using single synthetic nutrients delivered contradictive results in comparison to studies using fruits or vegetables (multinutrients).

Importantly, vitamins C and E also stimulate NO production thus protecting from endothelial dysfunction [30]. Reduced NO bioavailability leading to less scavenging of superoxide anions is another important factor for the control of vascular functionality and oxidative stress in obesity and MetS [31]. NO is produced from L-arginine by NO synthase (NOS) and an important cofactor of NOS stimulating the production of NO is BH4 [32]. Oxidation of BH4 into BH2 leads to disturbed NO metabolism, production of peroxynitrite, and deterioration of oxidative stress [33]. Here, we demonstrate in mice that BH4 oxidation occurs before other CVD risk factors develop making this an effective and easy to measure marker for early oxidative stress during the onset of obesity and MetS. If this is also true in humans needs to be evaluated in future clinical trials.

Corabion also contains EPA and DHA, polyunsaturated fatty acids that have vasoprotective, anti-inflammatory properties and are well known for lowering triglyceride levels [34]. In our experiments, we also observed significantly lower triglyceride levels and inflammation markers when combining the HFD with corabion (Figures 1(c) and 4). Thus, in combination with the NO source L-arginine, EPA and DHA within corabion are likely responsible for a vasoprotective effect by reducing triglyceride levels and supporting endothelial functionality, whereas vitamins C and E contribute to the beneficial effects of corabion by reverting the early oxidative stress induced by HFD. Obesity can increase the risk for CVD due to a chronic microinflammatory response leading to endothelial dysfunction [35]. Obesity-induced chronic microinflammation is characterized by increased production of proinflammatory cytokines such as IL6 that in turn induces CRP production in the liver and secretion into the blood [36–38]. Interestingly, we did not observe increased levels of TNF-*α* but increases in IL6 production in the kidney and CRP levels in the blood suggesting that HFD induces early signs of a systemic microinflammatory response already after 2 weeks. The exact temporal chain of events during development of obesity and obesity-related CVD is still not completely understood. However, while we already observed a slight increase in oxidative stress after 1 week and a strong one after 2 weeks and only a slight increase of inflammatory markers after 2 weeks, this suggests that oxidative stress precedes microinflammation and subsequent increases in blood pressure and serum triglyceride and glucose levels. Thus, corabion's main way of action is counteraction of HFD-induced oxidative stress and inflammatory responses that in turn prevent the development of other CVD risk factors during HFD. If oxidative stress is indeed the cause for inflammation needs to be carefully investigated in future time-course studies* in vivo*.

## 5. Conclusions

In conclusion, we found that the nutrient mixture corabion (but not its single components) is able to reverse the increases in CVD risk factors induced by HFD. Our data suggest that oxidative stress and microinflammation are the first risk factors to increase during HFD that precede the onset of hypertension and increases in serum triglyceride and glucose levels. Thus, we propose that measurement of the plasma BH4/BH2 ratio could be a convenient diagnostic marker of early obesity-induced CVD that will allow an early start of preventive treatments. In this respect, corabion may be a valuable diet supplement to prevent HFD-induced increases in oxidative stress, microinflammation, and thus CVD.

## Figures and Tables

**Table 1:** Data of control mice and mice on a high-fat diet (HFD) in combination with single component treatments and corabion treatment after 8 weeks.

Treatment	BH2 (pmol/mL)	BH4 (pmol/mL)	Body weight (g)	Blood glucose (mg/dL)	Triglycerides (mg/dL)
Control	7 ± 1	20 ± 3	27 ± 3	62 ± 4	142 ± 25
HFD	26 ± 4^*^	6 ± 1^*^	34 ± 2^*^	96 ± 8^*^	214 ± 35^*^
HFD + L-arginine	20 ± 3	7 ± 2	34 ± 3	90 ± 1	200 ± 21
HFD + vit. E	23 ± 4	9 ± 3	31 ± 2	98 ± 6	221 ± 15
HFD + vit. C	20 ± 4	5 ± 1	32 ± 1.5	92 ± 8	192 ± 29
HFD + corabion	12 ± 4^*^	16 ± 3^*^	33 ± 1.5	64 ± 6^*^	142 ± 28^*^

Data are presented as mean ± SDM; *n* = 5 per group. ^∗^ *P* < 0.05.

## Abbreviations

AOT: Antioxidative treatment
BH2: Dihydrobiopterin
BH4: Tetrahydrobiopterin
CVD: Cardiovascular diseases
DHA: Docosahexaenoic acid
DHE: Dihydroethidium
EPA: Eicosapentaenoic acid
HFD: High-fat diet
MetS: Metabolic syndrome
NO: Nitric oxide
ROS: Reactive oxygen species.
